# Supplementary material for: What determines the optimal pharmacological treatment of atrial fibrillation? Insights from in silico trials in 800 virtual atria
Source: J Physiol. 2023 Jul 20;601(18):4013–32. doi: 10.1113/JP284730 (PMC10952228; doi:10.1113/JP284730)
Supplement: Supplementary file 1 — Statistical Summary Document [file TJP-601-4013-s001.docx]

**Manuscript Title:** What determines the optimal pharmacological treatment of atrial fibrillation? Insights from in-silico trials in 800 virtual atria

**Authors:** Albert Dasí, Michael T.B. Pope, Rohan S. Wijesurendra, Tim R. Betts, Rafael Sachetto, Alfonso Bueno-Orovio, Blanca Rodriguez

**Underlying hypothesis:** This investigation tests the hypothesis that the ionic current distribution of the atria influences atrial fibrillation (AF) maintenance.

**Definitions of ‘n’:**

n = ionic current profiles yielding sustained AF

**Statistical summary table:**

| Variable Analysed | Finding/ conclusion | Sustained AF for (percentage) of LVA | Median | IQR | n val. | Units | Statistical test | Figure/ table in which data are presented |
| --- | --- | --- | --- | --- | --- | --- | --- | --- |
| Variation (%)  I_to_ density | Ionic current profiles yielding sustained AF for more LVA distributions have increased  I_to_ density | <25% LVA | -8 | 36 | 23 | % | Wilcoxon rank sum test | Figure 2 |
|  |  | 25-75% | -12 | 47 | 32 | % | Wilcoxon rank sum test | Figure 2 |
|  |  | >75% LVA | 2 | 46 | 45 | % | Wilcoxon rank sum test | Figure 2 |
| Variation (%)  I_K1_ density | Ionic current profiles yielding sustained AF for more LVA distributions have increased  I_K1_ density | <25% LVA | -13 | 34 | 23 | % | Wilcoxon rank sum test | Figure 2 |
|  |  | 25-75% | -12 | 36 | 32 | % | Wilcoxon rank sum test | Figure 2 |
|  |  | >75% LVA | 24 | 39 | 45 | % | Wilcoxon rank sum test | Figure 2 |
| Variation (%)  I_Ks_ density | Higher I_Ks_ is observed in models yielding AF in 25-75% LVA distributions but not >75% LVA | <25% LVA | 3 | 56 | 23 | % | Wilcoxon rank sum test | Figure 2 |
|  |  | 25-75% | 15 | 46 | 32 | % | Wilcoxon rank sum test | Figure 2 |
|  |  | >75% LVA | -12 | 42 | 45 | % | Wilcoxon rank sum test | Figure 2 |
| Variation (%)  I_CaL_ density | Ionic current profiles yielding sustained AF for more LVA distributions have decreased  I_CaL_ density | <25% LVA | 18 | 37 | 23 | % | Wilcoxon rank sum test | Figure 2 |
|  |  | 25-75% | -1 | 52 | 32 | % | Wilcoxon rank sum test | Figure 2 |
|  |  | >75% LVA | -22 | 36 | 45 | % | Wilcoxon rank sum test | Figure 2 |
| Variation (%)  I_NaK_ density | Ionic current profiles yielding sustained AF for more LVA distributions have increased  I_NaK_ density | <25% LVA | -29 | 31 | 23 | % | Wilcoxon rank sum test | Figure 2 |
|  |  | 25-75% | 5 | 43 | 32 | % | Wilcoxon rank sum test | Figure 2 |
|  |  | >75% LVA | 15 | 43 | 45 | % | Wilcoxon rank sum test | Figure 2 |

**Underlying hypothesis:** This investigation tests the hypothesis that higher I_K1_ is needed for AF maintenance as LVA increases in the left atrium.

n = ionic current profiles yielding sustained AF

**Statistical summary table:**

| Variable Analysed | Finding/ conclusion | Sustained AF for small vs. big  LVA extension | Median | IQR | n val. | Units | Statistical test | Figure/ table in which data are presented |
| --- | --- | --- | --- | --- | --- | --- | --- | --- |
| Variation (%)  I_K1_ density | Higher I_K1_ is observed in ionic current profiles sustaining AF in atria with higher left atrial LVA extension | <30% LVA extension left atrium | -31 | 29 | 9 | % | Wilcoxon rank sum test | Figure 2 |
|  |  | >40% LVA extension left atrium | -5 | 32 | 9 | % | Wilcoxon rank sum test | Figure 2 |

**Underlying hypothesis:** This investigation tests the hypothesis that higher I_K1_ is needed for AF maintenance as LVA increases in the left atrial posterior wall.

**Statistical summary table:**

| Variable Analysed | Finding/ conclusion | Sustained AF for small vs. big  LVA extension | Median | IQR | n val. | Units | Statistical test | Figure/ table in which data are presented |
| --- | --- | --- | --- | --- | --- | --- | --- | --- |
| Variation (%)  I_K1_ density | Higher I_K1_ is observed in ionic current profiles sustaining AF in atria with higher LVA extension in the left atrial posterior wall | <30% LVA extension left atrium | -34 | 20 | 11 | % | Wilcoxon rank sum test | Figure 2 |
|  |  | >40% LVA extension left atrium | -4 | 37 | 11 | % | Wilcoxon rank sum test | Figure 2 |

**Underlying hypothesis:** This investigation tests the hypothesis that higher I_K1_ and lower I_CaL_ increase the dominant frequency.

**Definitions of ‘n’:**

n = atria with sustained AF

**Statistical summary table:**

| Variable analysed | Finding/ conclusion | Scenario Analysed | Median | IQR | n val. | Units | Statistical test | Figure/ table in which data are presented |
| --- | --- | --- | --- | --- | --- | --- | --- | --- |
| AF dominant frequency for high  I_K1_ density | Higher I_K1_ density increases AF dominant frequency | Overall Population | 6.63 | 1.65 | 494 | Hz | Wilcoxon rank sum test | Figure 3 |
|  |  | High Ito, INaK, INa  High IK1 | 8.47 | 1.98 | 53 | Hz | Wilcoxon rank sum test | Figure 3 |
|  |  | High Ito, INaK, INa  Low IK1 | 6.78 | 0.62 | 17 | Hz | Wilcoxon rank sum test | Figure 3 |
| AF dominant frequency for low  I_CaL_ density | Lower I_CaL_ density increases AF dominant frequency | Overall Population | 6.63 | 1.65 | 494 | Hz | Wilcoxon rank sum test | Figure 3 |
|  |  | High Ito, INaK, INa, IK1  High ICaL | 8.91 | 1.46 | 32 | Hz | Wilcoxon rank sum test | Figure 3 |
|  |  | High Ito, INaK, INa, IK1  Low ICaL | 7.15 | 1.8 | 21 | Hz | Wilcoxon rank sum test | Figure 3 |

**Underlying hypothesis:** Atria responding to vernakalant have a longer refractoriness, due to elevated ICaL.

**Definitions of ‘n’:**

n = ionic current profiles

**Statistical summary table:**

| Variable analysed | Finding/ conclusion | Ionic current profiles responding to vernakalant in (%) of LVA distributions | Median | IQR | n val. | Units | Statistical test | Figure/ table in which data are presented |
| --- | --- | --- | --- | --- | --- | --- | --- | --- |
| Variation (%)  I_CaL_ density | Vernakalant is effective in atria with ICaL up-regulation | < 50% LVA distributions | -28 | 29 | 35 | % | Wilcoxon rank sum test | Figure 5 |
|  |  | 50 - 90% LVA distributions | -7 | 47 | 20 | % | Wilcoxon rank sum test | Figure 5 |
|  |  | 100 % LVA distributions | 15 | 40 | 33 | % | Wilcoxon rank sum test | Figure 5 |
| Variation (%)  I_NaK_ density | Vernakalant is effective in atria with INaK up-regulation | < 50% LVA distributions | 16 | 37 | 35 | % | Wilcoxon rank sum test | Figure 5 |
|  |  | 50 - 90% LVA distributions | 3 | 49 | 20 | % | Wilcoxon rank sum test | Figure 5 |
|  |  | 100 % LVA distributions | -1 | 43 | 33 | % | Wilcoxon rank sum test | Figure 5 |

**Underlying hypothesis:** Atria not responding to amiodarone have the highest excitability, due to ionic current dysregulation.

**Definitions of ‘n’:**

n = ionic current profiles

**Statistical summary table:**

| Variable analysed | Finding/ conclusion | Ionic current profiles responding to amiodarone in (%) of LVA distributions | Median | IQR | n val. | Units | Statistical test | Figure/ table in which data are presented |
| --- | --- | --- | --- | --- | --- | --- | --- | --- |
| Variation (%)  I_K1_ density | Amiodarone is effective in atria with IK1 down-regulation | < 50% LVA distributions | 27 | 58 | 19 | % | Wilcoxon rank sum test | Figure 5 |
|  |  | 50 - 90% LVA distributions | 12 | 56 | 20 | % | Wilcoxon rank sum test | Figure 5 |
|  |  | 100 % LVA distributions | -2 | 45 | 49 | % | Wilcoxon rank sum test | Figure 5 |
| Variation (%)  I_CaL_ density | Amiodarone is effective in atria with IK1 up-regulation | < 50% LVA distributions | -33 | 32 | 19 | % | Wilcoxon rank sum test | Figure 5 |
|  |  | 50 - 90% LVA distributions | -11 | 38 | 20 | % | Wilcoxon rank sum test | Figure 5 |
|  |  | 100 % LVA distributions | 5 | 47 | 49 | % | Wilcoxon rank sum test | Figure 5 |
| Variation (%)  I_NaK_ density | Amiodarone is effective in atria with INaK down-regulation | < 50% LVA distributions | 29 | 39 | 19 | % | Wilcoxon rank sum test | Figure 5 |
|  |  | 50 - 90% LVA distributions | 9 | 30 | 20 | % | Wilcoxon rank sum test | Figure 5 |
|  |  | 100 % LVA distributions | -2 | 46 | 49 | % | Wilcoxon rank sum test | Figure 5 |
| Variation (%)  I_NCX_ density | Amiodarone is effective in atria with INCX up-regulation | < 50% LVA distributions | 29 | 60 | 19 | % | Wilcoxon rank sum test | Figure 5 |
|  |  | 50 - 90% LVA distributions | -5 | 38 | 20 | % | Wilcoxon rank sum test | Figure 5 |
|  |  | 100 % LVA distributions | 0 | 50 | 49 | % | Wilcoxon rank sum test | Figure 5 |

**Underlying hypothesis:** Atria responding to flecainide have lower INa.

**Definitions of ‘n’:**

n = ionic current profiles

**Statistical summary table:**

| Variable analysed | Finding/ conclusion | Ionic current profiles responding to flecainide in (%) of LVA distributions | Median | IQR | n val. | Units | Statistical test | Figure/ table in which data are presented |
| --- | --- | --- | --- | --- | --- | --- | --- | --- |
| Variation (%)  I_Na_ density | Amiodarone is effective in atria with INa down-regulation | < 50% LVA distributions | 15 | 34 | 47 | % | Wilcoxon rank sum test | Figure 5 |
|  |  | 50 - 90% LVA distributions | -7 | 10 | 6 | % | Wilcoxon rank sum test | Figure 5 |
|  |  | 100 % LVA distributions | -26 | 26 | 35 | % | Wilcoxon rank sum test | Figure 5 |

**Underlying hypothesis:** The ionic current profile of the atria can guide optimal stratification of AF patients to pharmacological therapy.

**Definitions of ‘n’:**

n = ionic current profiles

**Statistical summary table:**

| Variable analysed | Finding/ conclusion | Ionic current profiles responding to | Median | IQR | n val. | Units | Statistical test | Figure/ table in which data are presented |
| --- | --- | --- | --- | --- | --- | --- | --- | --- |
| **Variation I_CaL_ density**  Vernakalant  vs  Amiodarone | Amiodarone is more effective than vernakalant in atria with lower **ICaL** | Vernakalant | 15 | 18 | 6 | % | Wilcoxon rank sum test | Figure 5 |
|  |  | Amiodarone | -17 | 42 | 22 | % | Wilcoxon rank sum test | Figure 5 |
|  |  | Both | 18 | 25 | 27 | % | Wilcoxon rank sum test | Figure 5 |
| **Variation I_Na_ density**  Amiodarone  vs  Flecainide | Amiodarone is more effective than flecainide in atria with higher **INa** | Amiodarone | 13 | 34 | 29 | % | Wilcoxon rank sum test | Figure 5 |
|  |  | Flecainide | -22 | 57 | 15 | % | Wilcoxon rank sum test | Figure 5 |
|  |  | Both | -32 | 20 | 20 | % | Wilcoxon rank sum test | Figure 5 |
| **Variation I_CaL_ density**  Vernakalant  vs  Flecainide | Flecainide is more effective than flecainide in atria with lower **ICaL** | Vernakalant | 5 | 39 | 17 | % | Wilcoxon rank sum test | Figure 5 |
|  |  | Flecainide | 27 | 23 | 19 | % | Wilcoxon rank sum test | Figure 5 |
|  |  | Both | -22 | 32 | 16 | % | Wilcoxon rank sum test | Figure 5 |
| **Variation I_Na_ density**  Vernakalant  vs  Flecainide | Vernakalant is more effective than flecainide in atria with higher **INa** | Vernakalant | 27 | 25 | 17 | % | Wilcoxon rank sum test | Figure 5 |
|  |  | Flecainide | -38 | 21 | 19 | % | Wilcoxon rank sum test | Figure 5 |
|  |  | Both | -3 | 63 | 16 | % | Wilcoxon rank sum test | Figure 5 |
